# The Influence of Plants from the *Alliaceae* Family on Morphological Parameters of the Intestine in Atherogenic Rats

**DOI:** 10.3390/nu13113876

**Published:** 2021-10-29

**Authors:** Katarzyna Najman, Hanna Leontowicz, Maria Leontowicz

**Affiliations:** 1 Department of Functional and Organic Food, Institute of Human Nutrition Sciences, Warsaw University of Life Sciences, Nowoursynowska 159c, 02-776 Warsaw, Poland; 2 Department of Physiological Sciences, Institute of Veterinary Medicine, Warsaw University of Life Sciences, Nowoursynowska 159, 02-776 Warsaw, Poland; hanna_leontowicz@sggw.edu.pl (H.L.); maria_leontowicz@sggw.edu.pl (M.L.)

**Keywords:** garlic (*Allium sativum* L.), onion (*Allium cepa* L.), Wistar rat, cholesterol, atherosclerosis, ileum, villi length, tunica mucosa, tunica muscle

## Abstract

Bulbs from the *Alliaceae* family have been well-known and valued spices for thousands of years, not only for their unique flavor and aroma features, but also for their high nutritional and health-promoting values. Long-term or excessive consumption of these vegetables, especially raw garlic, can have side effects in the body (including in the digestive tract), causing a number of pathological changes in the intestinal wall; these changes lead, in turn, to its damage, dysfunction, and disorder development. Therefore, the aim of this study was to investigate the effect of the addition of freeze-dried vegetables from the *Alliaceae* family, i.e., garlic (*Allium sativum* L.), white onion, and red onion (*Allium cepa* L.) on the morphometric parameters (intestinal villi length, crypt depth, thickness of tunica mucosa, and the thickness of tunica muscle) of the jejunum of rats fed a semi-synthetic atherogenic diet (1% dietary cholesterol). In freeze-dried vegetables administered to rats, the contents of selected bioactive ingredients and their antioxidant potentials were determined. The effect of the onion vegetable supplements on growth parameters, serum lipid profile, plasma antioxidant potential, and the intestinal morphological parameters of rats loaded with cholesterol was determined. In an animal experiment, 30 male Wistar rats were divided into 5 diet groups, diet consumption and FER were studied. Supplementation of the atherogenic diet with vegetables improved the blood plasma lipid profiles and atherogenic indices, in a manner that was dependent on the type of supplementation used, with the best hypolipidemic and anti-atherosclerotic effects found in garlic use. The atherogenic diet, as well as the supplementation of this diet with the tested vegetables from the *Alliaceae* family, influenced the histological changes in the epithelium of the jejunum of rats. The damage to the intestinal mucosa was the greatest in animals fed an atherogenic diet supplemented with garlic. Bearing in mind that the desired beneficial therapeutic or prophylactic effects of onion vegetables (in particular garlic) in the course of various metabolic ailments (including atherosclerosis) are achieved during long-term supplementation, it is important to remember their possible cytotoxic effects (e.g., on the digestive tract) in order to achieve real benefits related to the supplementation with vegetables from the *Alliaceae* family.

## 1. Introduction

Due to their flavor and their high content of bioactive compounds, plants from the *Alliaceae* family—such as garlic (*Allium sativum* L.) and white and red onions (*Allium cepa* L.)—have been a common and integral part of diets for thousands of years, consumed in almost all cultures and cuisines of the world. They have been consumed both in the form of fresh, raw food additives and as an ingredient in many processed, ready-to-eat dishes [1,2].

Research on the health-promoting properties of garlic (*Allium sativum* L.) and onions (*Allium cepa* L.) proves that these plants have high contents of biologically active compounds and a high antioxidant potential, thanks to which, as a dietary component, they play an important role in the prevention and treatment of many diseases [3,4,5,6,7,8,9,10,11]. Due to their effective action in the prevention of civilization diseases, the interest of researchers in these plants is constantly growing [11,12] and they are perceived to be an important component of the diet [13]. In the last 25 years, many studies have focused on the role of *Alliaceae* bulbs in the prevention and treatment of cardiovascular diseases [12,13,14,15]. The formation and development of atherosclerotic lesions is fostered by many risk factors, the most important of which are blood lipids, increased platelet aggregation activity, and oxidative stress [16]. Lowering the content of triacylglycerols (TG), total cholesterol (TC) and its LDL-C fraction (LDL—low-density lipoprotein cholesterol), as well as increasing the antioxidant potential in the body—protecting the LDL-C fraction against oxidation—play an important role in the prevention of atherosclerosis and cardiovascular diseases [17].

The protective effect of garlic in atherosclerosis is attributed to its lipid-reducing properties and its direct anti-atherogenic effects in the arterial walls [18,19,20,21,22,23]. Research shows that garlic has great potential to inhibit platelet aggregation [13,19,20,22,24]. It reduces the formation of thromboxane and inhibits the activity of phospholipase and lipoxygenase that are produced in platelets [25]. The ajoenes contained in garlic strongly inhibit the metabolism of arachidonic acid through cyclooxygenase and lipoxygenase, thus limiting the synthesis of thromboxane A2 and 12-HETE (12-HETE—12-Hydroxyeicosatetraenoic acid) [13]. Studies on the hepatocytes of rats and humans have shown that garlic and its compounds may play a key role in inhibiting the activity of enzymes involved in the synthesis of cholesterol and fats [26,27]. Garlic lowers the activity of liver enzymes involved in the synthesis of lipids and cholesterol, such as apple enzyme, fatty acid synthase, glucose-6-phosphate dehydrogenase, and 3-hydroxy-3-methylglutaryl-coenzyme A (HMG-CoA) reductase [27,28,29].

The health-promoting effect of garlic is related to its vitamins (C and B), mineral compounds (selenium, calcium, potassium, and copper), and polyphenols, among other attributes, which, together with organic sulfur compounds, contribute to a high antioxidant potential [30,31,32,33,34,35,36,37,38,39,40]. The antioxidant effect of garlic is one of its most important health-promoting properties [41,42,43]. Studies have shown that garlic can protect blood vessels against the negative effects of free radicals by showing antioxidant activity [7,18,19,22,33]. Therefore, it protects the LDL-C fraction against oxidation, thus delaying the development of atherogenic changes and lowering the level of cholesterol and lipids in the blood [18,19,20,22,23,44]. By “scavenging” free radicals, it prevents lipid peroxidation and oxidative modification of LDL-C lipoproteins in vitro [45], which may be one of the mechanisms of garlic’s protective effect in atherosclerosis [18,19,22,23,46].

The health-promoting effect of onions in diseases of the cardiovascular system also results from its strong antioxidant properties [4,5,6,7,10,23,40,47,48]. Onion, like garlic, contains inhibitors of lipoxygenase and cyclooxygenase (enzymes that produce inflammatory hormones—prostaglandins and thromboxanes), which, together with the other biologically active compounds contained in it, exert an anti-inflammatory effect in the body [49]. Onions are rich in many biologically active compounds that reduce the risk factors for cardiovascular disease [5,6,8,10,11,22,50,51,52]. Epidemiological studies show that there is a strong association between a diet high in onion and a reduction in the risk of mortality from ischemic heart disease [3]. Consuming onion and onion extracts has been shown to lower blood lipid levels (total cholesterol, LDL-C, and triacylglycerols) in the blood [22,23,53,54].

It has also been found that consumption of onions inhibits cholesterol synthesis [55] and improves lipid metabolism [56]. In vitro studies have shown that raw onions have a high ability to reduce platelet aggregation [47,48,57], and this effect is greater for the consumption of spicy onions varieties [50]. Onions’ anti-aggregation properties are attributed to quercetin and cysteine sulfoxides, but the exact mechanism of their action is unknown. These compounds can stimulate the release of arachidonic acid, which initiates the metabolism of eicosanoids in mammals, leading to inhibition of thromboxane A synthesis that reduces platelet aggregation [58]. In pig studies, it was found that feeding for 6 weeks with fresh onions did not affect platelet aggregation but resulted in a significant reduction of triacylglycerols in the blood. The consumption of onions by these animals corresponded to the consumption of one onion per day by humans [53].

The available literature also includes studies on the adverse effects of the use of garlic in the diet, especially long-term supplementation or its excessive consumption [59], which may lead to inflammation and erosion of the gastric mucosa, atrophy of intestinal villi [60], impairment or inhibition of absorption in the small intestine [61], and increased concentration and activity of tissue enzyme ACP (ACP—acid phosphatase), ALP (ALP—alkaline phosphatase), and LDH (LDH—lactate dehydrogenase) in the small intestine [62] or in the blood serum of rats [59]. This can have a detrimental effect on the functions of the gut, leading to a number of gastrointestinal disorders. When used in excess, garlic may increase the activity of liver enzymes, both hepatotoxic and cytotoxic, not only on liver cells [63,64], but also in lung, heart, intestine, and stomach cells [65]. Such an increase can even result in the death of the animals (caused by gastric lesions, according to a rat study) [66]. However, the literature lacks data on the adverse effects of white and red onions, regarding the context of the negative effects on the gastrointestinal tract that are listed for garlic.

Research conducted so far on the role of garlic and onions in the prevention and alleviation of the course of diseases and ailments of the digestive tract is apparently insufficient, inconsistent, incomplete, and often contradictory. Moreover, they relate to various forms of garlic and onion use (fresh vegetables, fresh extract, freeze-dried forms, oil, and isolated compounds), supplementation time and dose, and finally various animal research models (e.g., rats [67,68,69,70,71,72,73], mice [74], pigs [75], broilers, and chickens [76,77,78,79]) with induced injuries of the gastrointestinal tract [67,69,71,73,74] or infected with pathogenic microorganisms [74]. In the available literature, no information was found on the effect of the addition of raw *Alliaceae* plants, administered in an atherogenic diet, on morphological changes in the intestine. This study assessed the effect of the addition of raw *Alliaceae* plants (i.e., garlic (*Allium sativum* L.), and white and red onion (*Allium cepa* L.)) in rats fed a semi-synthetic diet without cholesterol (or with its 1% share on growth); therefore, the use of diet, the atherogenic indices of blood serum, the antioxidant potentials of plasma, and morphological changes of the jejunum in Wistar rats were studied.

## 2. Materials and Methods

### 2.1. Plant Material

In this study, raw Polish plants from the *Alliaceae* family—i.e., garlic (*Allim sativum* L.) (“Harnaś” cultivar), white and red onion (*Allium cepa* L.) (“Amstrong” and “Red Baron” cultivar, respectively)—were collected from the test plots, which were donated by the Polish company Elena (Kokanin, Żelazków, Poland). The bulbs were washed, cleaned, peeled, cut into small, equal sized pieces and weighed. After that, the samples were frozen in liquid nitrogen and then freeze-dried in a freeze dryer (CHRIST ALPHA 2-4 LDplus Freeze Dryer, Martin Christ Gefriertrocknungsanlagen GmbH, Osterode am Harz, Germany), at a chamber pressure of 10 Pa, a drying chamber temperature of −50 °C, and a shelf temperature of 21 °C for 72 h. The freeze-dried material was weighed, ground to a powder in a laboratory knife mill (Grindomix GM 200, Retsch GmbH, Haan, Germany), packed into Falcone tubes and stored in freezing conditions at −20 °C until the commencement of analysis and animal study. Lyophilized material was used for testing the content of selected bioactive ingredients, their antioxidant potential, and for the animal studies.

#### 2.1.1. The Content of Bioactive Ingredients in Plants from the Alliaceae Family

##### Extracts Preparation

Defatted and deproteinized with acetonitrile (Pol-Aura Chemical Reagents, Zabrze, Poland), 100 mg of the freeze-dried plant material was washed with dichloromethane (Pol-Aura Chemical Reagents, Zabrze, Poland). Then, 50 mg of the sample was weighed into a tube with a screw cap and 5 mL of the extraction mixture (1.2 M HCl in 50% methanol) (Pol-Aura Chemical Reagents, Zabrze, Poland) was added. The samples were vortexed (Wizard Advanced IR Vortex Mixer, VELP Scientifica Srl, Usmate, Italy) for 1 min and then incubated (IKA KS 4000 Control, IKA^®^ Poland Ltd., Warsaw, Poland) for 3 h at 90 °C with shaking performed every 30 min. After incubation the samples were cooled, then diluted to 10 mL with methanol and centrifuged (MPW-380R, MPW Med. Instruments, Poland, Warsaw) (10 min, 5 °C, 6000 rpm). The obtained clear supernatants were used for the determination of bioactive ingredients and antioxidant activity.

##### Total Polyphenols Content

The total polyphenols content was determined according to the Singleton and Rossi [80] method, using the Folin–Ciocalteu reagent (Sigma-Aldrich, Poznań, Poland). The absorbance was measured using a spectrophotometer (UV–VIS UV-6100A, Metash Instruments Co., Ltd., Shanghai, China) at the wave-length of λ = 365 nm. Gallic acid (Sigma-Aldrich, Poznań, Poland) was used as a standard. The results were expressed as mg gallic acid equivalent per 1 g of dry matter (mg GAE/g d.m.). The determinations were performed in six independent replications.

##### Flavonoids Content 

According to the method of Singleton et al. [81], the flavonoids contents were extracted with 5% NaNO_2_, 10% AlCl_3_·6H_2_O and 1M NaOH (Pol-Aura Chemical Reagents, Zabrze, Poland). The absorbance was measured using a spectrophotometer (UV–VIS UV-6100A, Metash Instruments Co., Ltd., Shanghai, China) at the wave-length of λ = 510 nm. (+)-Catechin (Sigma-Aldrich, Poznań, Poland) was used as a standard. The results were expressed as the mg catechin equivalent, per 1 g of dry matter (mg CE/g d.m.). The determinations were performed in six independent replications.

##### Flavanols Content

The total flavanols content was determined according to the Feucht and Polster [82] method, using the DMACA (p-dimethylaminocinnamaldehyde) reagent (Sigma-Aldrich, Poznań, Poland). The absorbance was measured using a spectrophotometer (UV–VIS UV-6100A, Metash Instruments Co., Ltd., Shanghai, China) at the wave-length of λ = 640 nm. (+)-Catechin (Sigma-Aldrich, Poznań, Poland) was used as a standard. The results were expressed as the µg catechin equivalent, per 1 g of dry matter (µg CE/g d.m.). The determinations were performed in six independent replications.

##### Anthocyanins Content

The total anthocyanins content was determined according to the differential method of Lo Scalzo et al. [83]. The absorbances for the two pH values (1 and 4.5) were measured in a Beckman spectrophotometer (Beckman DU520 UV/VIS, Beckman Coulter Ltd., Warsaw, Poland) at a wave-length of λ = 510 nm. Cyanidin-3-glucoside (CGE) (Sigma-Aldrich, Poznań, Poland) was used as a standard. The results were expressed as the mg cyanidin-3-glucoside equivalent, per 1 kg of dry matter (mg CGE/kg d.m.). The determinations were performed in six independent replications.2.1.2. Antioxidant Activity in Plants from the Alliaceae Family

The antioxidant activity was determined according to the method of Ozgen et al. (2006), with free radical ABTS (2,2’-Azino-bis-(3-ethyl-benzothiazoline-6-sulfonic acid) diammonium salt) (Sigma-Aldrich, Poznań, Poland). The solution of the radical ABTS^•+^ was prepared by the interaction of ABTS and K2S2O8 (Sigma-Aldrich, Poznań, Poland) (7 mM/L ABTS, 2.45 mM/ L K_2_S_2_O_8_) and incubated at room temperature in the dark for 24 h. The ABTS^•+^ solution was then diluted with methanol until an absorbance of 0.7 was obtained at a wave-length λ = 734 nm. To a 10 µL sample of the extract (0.2 mg/L), 990 µL of ABTS^•+^ solution was added, and then the absorbance was measured in a spectrophotometer (UV–VIS UV-6100A, Metash Instruments Co., Ltd., Shanghai, China) at the wave-length of λ = 734 nm after exactly 6 h of incubation. Trolox (Sigma-Aldrich, Poznań, Poland) was used as a standard. The results were expressed as the µM Trolox equivalent, per 1 g of dry matter (µM TE/g d.m.). The determinations were performed in six independent replications.

### 2.2. Animals

The study was conducted in accordance with The Animal Care Committee of the Warsaw Agricultural University (Acceptation of II Local Commity for Etics in Animal Research in Warsaw), Warsaw, Poland. Male Wistar rats were purchased from Breeding of Laboratory Animals, Brwinów, Poland (veterinary identification number 14216203). 

#### 2.2.1. Experimental Design

The mean weight of the male Wistar rats (*n* = 30) at the beginning of the experiment was 121.8 ± 9.9 g. Rats were divided into five diet groups, each with 6 rats, these were named the following: control (C), cholesterol control (CH), cholesterol + garlic (CHG), cholesterol + onion white (CHOW), and cholesterol + onion red (CHOR). For the first 5 days of investigations, the animals underwent an adaptation period—all rats were fed the semi-purified control diet (C), which included wheat starch, casein, soybean oil, cellulose, vitamin (AIN-93-VX vitamin mix catalog no. 960402), and mineral (AIN-93-MX mineral mix catalog no. 960400) mixtures from the American Institute of Nutrition for laboratory animals. The rats were housed in metabolic cages (TECNIPLAST S.p.A., Buguggiate, Italy), in standard laboratory conditions as follows: an air-conditioned room, average air temperature 24 °C ± 0.5 °C, air humidity at 50%, and a diurnal light cycle of 12 h/12 h with lighting at 118 lx. During the 28 days of the experiment, the rats from the control group (C) were fed only a control diet (C), and the cholesterol-control group (CH) was fed a control diet with 1% of non-oxidized cholesterol (CH) of analytical grade (Sigma Chemical Co., St Louis, MO, USA). The rest of the animals received a cholesterol-containing diet, supplemented with lyophilized raw garlic (CHG), white onion (CHOW), and red onion (CHOR) in quantities that depended on the growing body weight of the animals during the experiment. These varied from 25 mg/day/rat (at the beginning) to 45 mg/day/rat (at the end of the experiment), which corresponded to 500 mg of lyophilized raw garlic/onion per 1 kg of body weight. The cholesterol batches were mixed carefully with the C diet (1:99) just before feeding the rats. All animals were fed the isocaloric diet once a day at 10.00 a.m. ad libitum and they had unrestricted access to drinking water. Feed intakes were monitored daily, and body weights were measured weekly.

#### 2.2.2. Animal Sacrifice

After the last administration (24 h) and overnight starvation, the rats were sacrificed using Halothane (Narcotan, Zentiva Poland Ltd., Warsaw, Poland): the blood samples were taken from the left atrium from the heart; plasma and serum were prepared and used for a wide range of laboratory tests; organs (heart, spleen, kidney, liver, and aorta) were taken to the histopathological examination; and samples of ileum were collected for morphometric evaluation. In the following part of the study, the morphometrical and histological characteristics of ileum (villi length, crypt depth, and the thickness of tunica mucosa and muscle) of experimented rats are presented. 

#### 2.2.3. Sampling and Measurements

##### Basic Experimental Data

Body weights (BW) were recorded at the beginning of the experiment, weekly during the experiment, and at the end of the experiment. Feed intake (FI) and the intake of lyophilized vegetables additions (garlic, white onion, and red onion) were monitored every day. Feed efficiency ratio (FER) was calculated as a gram of feed consumed, per gram of body weight gain (g/g). Somatic index of liver (SI-L) was calculated as a ratio of liver weight to final body weight and was expressed as a percentage of body weight (% BW).

##### Biochemical Analysis

The tests included determination of plasma lipids: total cholesterol (TC), low-density lipoprotein cholesterol (LDL-C), high-density lipoprotein cholesterol (HDL-C), triglicerides (TG), and plasma antioxidant activity (ABTS). A Siemens analyzer (ADVIA^®^ 1650 Chemistry System from Bayer^®^ Clinical Methods, Labexchange, Die Laborgerätebörse GmbH, Burladingen, Germany) was used in the determination of the serum lipid profile. Atherogenic indices: atherogenic index (AI) (calculated as LDL-C/HDL-C), Castelli’s atherogenic index (CAI) (calculated as a TC/HDL-C), and the atherogenic index of plasma (API) (calculated as log(TG/HDL-C)) of the experimented upon rats are presented. 

##### The Antioxidant Potential of Plasma

The antioxidant activity was determined according to the method of Ozgen et al. [84], with free radical ABTS (2,2’-Azino-bis-(3-ethyl-benzothiazoline-6-sulfonic acid) diammonium salt) (Sigma-Aldrich, Poznań, Poland), in the same way as described above for plant material extracts. Trolox (Sigma-Aldrich, Poznań, Poland) was used as a standard. The results were expressed as the mM trolox equivalent per 1 L of plasma (µM TE/L) The determinations were performed in six independent replications.

##### Tissue Sampling Procedure for the Ileum Histology

After 28 days of experiment, all rats were euthanized, and the samples of ileum were collected for morphometric evaluation of villi length, crypt depth, and the thickness of tunica mucosa and muscle. Tissues for histological preparations (segments of the intestine) were gently rinsed with physiological saline (0.9% NaCl, Pol-Aura Chemical Reagents, Zabrze, Poland) to remove any residual digestive contents and fixed in 10% buffered formalin saline (pH = 7.2) (Chempur^®^, Piekary Śląskie, Poland). The tissues were dehydrated in a graded series of ethanol (Pol-Aura Chemical Reagents, Zabrze, Poland), infiltrated with xylene (Pol-Aura Chemical Reagents, Zabrze, Poland) and liquid paraffin (Pol-Aura Chemical Reagents, Zabrze, Poland) in carousel tissue processor (STP 120 Spin Tissue Processor, Especialidades Médicas MYR, S.L., Lleida, Spain). They were then embedded in paraffin wax using a modular tissue embedding center (MICROM EC350-2, MICROM, Thermo Fisher Scientific Inc., Waltham, MA, USA), and cut at 5 µm thick sections using a rotary microtome (MICROM Section Transfer System HM325, MICROM, Thermo Fisher Scientific Inc., Waltham, MA, USA).

The paraffin sections (5 µm) were placed on glass slides and dried in a laboratory thermostat (WAMED SSP, Warsaw, Poland) at 40 °C for 12 h. After that, samples were prepared according using the routine histological methods for light microscopy, stained with hematoxylin-eosin (HE) (Pol-Aura Chemical Reagents, Zabrze, Poland), and closed in a Roti^®^-Histokitt (Carl Roth, Linegal Chemicals Ltd., Warsaw, Poland).

##### Light Microscope Examination

The tissue section images were evaluating using a light microscope (OLYMPUS BX61, Olympus Poland Ltd., Warsaw, Poland) coupled with camera and computer software (Cell^P 3.2. imaging software, Olympus, Hamburg, Germany). In every intestinal slide, the height of six well-oriented villi and the depth of six corresponding intestinal crypts, the thickness of tunica mucosa and muscle—selected randomly from six different regions of each sampled tissue—were measured under the light microscope, which was fitted with a stage micrometer. From the results obtained, the average values for each rat and experimental group were calculated. 

### 2.3. Statistical Analysis

The results of this study are shown as means and are accompanied by standard deviation values (±SD) of six measurements. One-way analysis of variance (ANOVA) was appropriate for the statistical evaluation of results, following Duncan’s multiple-range tests to assess differences between groups’ means, we used the statistical software package Statistica 13.0 (Tibco Software Inc., Palo Alto, CA, USA). The *p*-values of < 0.05 were considered to be significant.

## 3. Results and Discussion

### 3.1. Plant Material

The contents of selected bioactive ingredients (total polyphenols, flavonoids, flavanols, and anthocyanins) and the antioxidant activity of freeze-dried plants from the Alliaceae family (garlic, white onion, and red onion) are presented in Table 1.

According to the results from the research, the onion plants differed in terms of the total polyphenol content: the highest content of these components was found in red onion (29.7 ± 1.3 mg GAE/g d.m.), and the content was lower in white onion and in garlic, by 16 and 65%, respectively. These results are comparable with data in the literature, in which the content of these bioactive ingredients for various onion varieties ranged from 10.6 to 21.2 mg GAE/g d.m. [85], and for garlic, there was an average of 18.9 mg GAE/g d.m. [86,87]. Moreover, other authors obtained similar results for white and red onions [88,89,90]. Prakash et al. [89] showed a higher content of total polyphenols in various onion cultivars (4.6–74.1 mg GAE/g d.m.) and found the highest content of these compounds in the external dry husk of red onion compared to white onion. Their research indicates that the content of these compounds varies in different parts of tubers, independent of the onion cultivars (white, yellow, purple, or red); additionally, the content changes in a similar direction—inner <middle <outer layers—which is related, inter alia, with the varying exposure of these tissues to light.

According to the literature data, flavonoids are present in greater amounts in white onion varieties [6,51,91], which is consistent with the results obtained in the present study. The content of flavonoids (Table 1) in garlic was (*p < 0.05*) the lowest among the studied bulbs (amounting to an average of 3.4 ± 0.3 mg CE/g d.m.) and was significantly higher in red onions (3.8 ± 0.3 CE/g d.m.) and in white onions (4.0 ± 0.3 CE/g d.m.). Higher results were obtained by Lin and Tang [88]. Similar results, but for different bulb varieties, were obtained by Sellapapan and Akoh [85]. According to other authors, these values may be even higher and change depending on the cultivar [92] and growing conditions [93] in the direction from red to white onion.

The content of flavanols in the studied bulbous plants ranged from 4.9 ± 0.4 to 6.7 ± 0.4 µg CE/g d.m. (for white onion and garlic, respectively) and was higher than the values obtained by Ninfali et al. [94]. According to De Pascual-Teresa et al. [95], the content of flavanols in vegetables ranges from completely undetectable amounts to 184 mg/100 g d.m.

Among the examined bulbs, no anthocyanins were found in garlic, but their content in red onion amounted to 460.2 ± 10.9 mg CGE/kg d.m. and was over 16 times higher than in white onion (28.3 ± 1.3 mg CGE/kg d.m). The results obtained were similar to those reported by Slimestad et al. [92]. The high content of these bioactive ingredients gives the red onion its characteristic dark red or purple color, and at the same time provides a high antioxidant potential [96].

The differences in the contents of biologically active compounds in vegetables are reflected in the different values of their antioxidant potential. Some authors suggest that a higher content of polyphenolic compounds with strong antioxidant properties is accompanied by a higher antioxidant potential of vegetables [4,6,7,37,42,43,51,85,97]. Many researchers have found a strong linear relationship between the antioxidant potential and the content of polyphenols (correlation coefficient 0.96) [86,87,97,98,99]. This was confirmed by the research carried out in this study. The antioxidant potential of the studied bulbs showed the highest value in red onion, but was significantly lower in white onion and in garlic (on average, 57.6 ± 7.0, 44.8 ± 6.7, and 36.5 ± 6.5 µM TE/g d.m., respectively).

### 3.2. Animal Study

In the in vivo studies conducted, the effect of the addition of freeze-dried vegetables (garlic, white onion, and red onion) on the physiological parameters of rats fed a semi-synthetic and/or atherogenic diet (with 1% cholesterol) was assessed. The results regarding feed, cholesterol, and lyophilized vegetables intake are presented in Table 2, and the results of rat growth parameters, including body weight gain, feed efficiency factor (FER), and somatic index of liver (SI-L) after 28 days of experiment, are presented in Table 3.

As can be seen from the data in Table 2, the mean feed intake of all experimental animals was 533.0 ± 57.4 g, with the (*p* < 0.05) highest reported in animals that were fed the control diet (C) without the addition of cholesterol and freeze-dried vegetables (589.8 ± 68.4 g), as well as in the CHOR group. The addition of cholesterol (CH) reduced consumption by about 15%. Animals from the groups that were fed an atherogenic diet supplemented with freeze-dried vegetables showed significant (*p* < 0.05) differences in feed intake, depending on the type of supplementation used. The addition of freeze-dried garlic used in the atherogenic diet (CHG) reduced its consumption to the greatest extent (480.9 ± 42.0 g), while the highest consumption of the atherogenic diet was found in the group of animals supplemented with freeze-dried red onion (CHOR) (578.9 ± 16.3 g). At the same time, in this group of animals (CHOR), the highest consumption of cholesterol and the freeze-dried vegetable supplement (5.8 ± 0.2 g and 700.1 ± 23.2 g, respectively) was recorded during the 28 day experiment.

The differences in dietary consumption in the experimental animals were reflected in the body weight gain, the feed efficiency (FER), and the somatic index of liver (SI-L) during the 28 day experiment (Table 3). The significantly highest (*p* < 0.05) body weight gain was found in animals that were fed with the control diet (C)—where it amounted to 178.2 ± 27.0 g—and the supplementation of cholesterol (1% in diet) significantly decreased this parameter (134.9 ± 26.2 g). Animals consuming the atherogenic diet supplemented with freeze-dried vegetables showed differences in body weight gain, with the lowest gains in the group of animals fed the atherogenic diet with freeze-dried garlic (113.9 ± 27.0 g), and the highest gain in the group supplemented with red onion (CHOR) (175.4 ± 17.8 g).

The experiment also showed significant (*p* < 0.05) differences in the feed efficiency ratio (FER) depending on its group. The most favorable FER was found in the control group (C), devoid of cholesterol (3.3 ± 0.3 g/g), and in the atherogenic group supplemented with red onion (3.3 ± 0.3 g/g). The animals that were fed the atherogenic diet with the addition of freeze-dried garlic used the diet the least (4.3 ± 0.5 g/g).

There were no significant (*p* < 0.05) differences between the groups receiving the control and the atherogenic diet (with 1% cholesterol) for the somatic index of the liver (SI-L); however, the percentage of the weight of this organ in the overall body weight of rats fed the atherogenic diet was slightly higher (3.7 ± 0.4%) than in the control group (3.6 ± 0.4%), which was due to high cholesterol consumption (5.0 ± 0.4 g) in these animals. The highest somatic liver index (SI-L) was found in rats receiving an atherogenic diet with the addition of freeze-dried red onion (CHOR) (3.7 ± 0.4%), for which cholesterol consumption was the highest (5.8 ± 0.2 g). The lowest SI-L was found in the group of animals supplemented with garlic (3.3 ± 0.3%), for which the cholesterol consumption during the 28 day experiment was the lowest (4.8 ± 0.4 g), but these observed differences were not statistically significant. According to the research of Basciano et al. [100], the accumulation of fat in the liver results in an increase in the mass of this organ, and one of the causes of fatty liver is increased lipogenesis.

The results obtained in this study are confirmed by the studies of other authors. In a similar, although longer (6 weeks) experiment on rats, Gorinstein et al. [23] demonstrated a higher degree of fatty liver and a higher concentration of lipids in the liver (349.7 ± 28.1 µg/µm^3^) in rats fed an atherogenic diet with cholesterol addition compared to the control group (without cholesterol) (95.1 ± 6.4 µg/µm^3^). Additionally, the addition of onion vegetables in the atherogenic diet improved the lipid indexes of the liver, with the best effect found in the group of animals receiving the addition of garlic (148.8 ± 10.7 µg/µm^3^). Moreover, Mahfouz and Kummerow [101] and Lawson and Gardner [102] showed a clearly lower concentration of lipids and cholesterol in the liver in groups of animals that were fed garlic powder. A similar relationship was found by Zeng et al. [103] in an experiment on mice fed a high cholesterol diet enriched with garlic oil.

**Figure 1:**
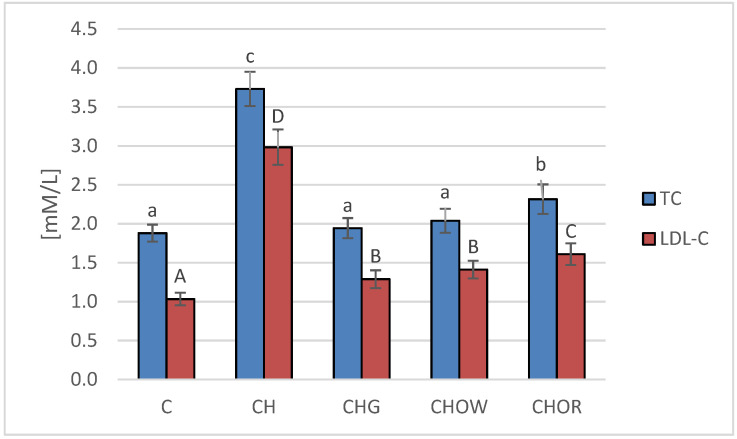
Changes in the plasma lipid profile (TC and LDL-C) in rats fed control and cholesterol-containing (1% of cholesterol) diets, supplemented with lyophilized vegetables (garlic, white onion, and red onion) during the 28 day experiment. Values are means of 6 measurements ± SD. Means in columns with different superscripts letters in common differ significantly (*p* < 0.05): a–c indicate values for TC and A–C indicate values for LDL-C. Abbreviations: C—control group; CH—control group with 1% of cholesterol; CHG—group with 1% of cholesterol and lyophilized garlic addition; CHOW—group with 1% of cholesterol and lyophilized white onion addition; CHOR—group with 1% of cholesterol and lyophilized raw red onion addition; TC—total cholesterol, LDL-C—low-density lipoprotein cholesterol fraction.

In the 28 day experiment we conducted, the effects of dietary nutrition—with or without cholesterol, and through the supplementation of an atherogenic diet with freeze-dried onion vegetables—on the lipid profile of rats’ plasma was assessed, and the results obtained are presented in Figure 1.

The highest concentration of total cholesterol (TC) and LDL cholesterol fraction (LDL-C) in blood serum was found in rats fed a diet with 1% cholesterol (3.7 ± 0.2 and 3.0 ± 0.2 mM/L, respectively). It was significantly higher compared to the control group (C) that was fed with a diet without it (1.9 ± 0.1 and 1.0 ± 0.1 mM/L, respectively). The addition of freeze-dried vegetables to the atherogenic (CH) diet significantly improved the lipid profile of rats’ blood serum, and these changes largely depended on the type of additive used. The most favorable effect was found in the group supplemented with garlic (CHG), in which the concentrations of TC and LDL-C decreased by approx. 48 and 56%, on average, respectively, in relation to the atherogenic (CH) diet. A slightly weaker effect was found in the case of the addition of freeze-dried white onion (CHOW), where the TC content in rats’ serum decreased by approx. 45%, and LDL-C by approx. 53%. The weakest effect was characteristic for the freeze-dried red onion (CHOR), the addition of which to an atherogenic diet resulted in a decrease in TC and LDL-C concentrations, by approx. 38 and 46%, on average, respectively. In the case of the HDL-C fraction, there were no statistically significant differences between groups C and CH and those receiving the addition of freeze-dried onion vegetables in the atherogenic diet, the mean concentration of HDL-C fraction in all animals was 0.7 ± 0.1 mM/L, with the highest in animals fed a diet without cholesterol (C) (0.8 ± 0.2 mM/L).

The results obtained in this study were confirmed in the studies of other authors, but they assessed the effects of various preparations made of garlic or onion, or pure compounds isolated from vegetables (e.g. quercetin) [18,19,22,26,27,36,44,55,57]. The available literature shows that garlic used in rats with hypercholesterolemia caused by high-cholesterol diets significantly (*p* < 0.05) lowered the concentration of total cholesterol, triacylglycerols, and LDL-Cs [18,19,22,33,104,105,106,107], without a significant effect on the proportion of HDL-C fraction. 

In this study, no changes were found in the share of this fraction in the blood. Some studies suggest that a significant reduction in the content of total cholesterol, LDL-C fraction, and triacylglycerols is accompanied by an increase in HDL-C levels, which is demonstrated in an experiment on rats by Aouadi et al. [108], among others. No such relationship was found in this study. The studies of other authors also confirm the beneficial effect of onions on the lipid profile. Onion decreased both the content of total cholesterol, its LDL-C fraction, and triacylglycerol in rats, hamsters [109,110], and pigs [53,56]. 

Taking into account the similarity of the biologically active compounds that are present in garlic and onions—as well as the fact that the subsequent stages of synthesis, absorption, or transformation of cholesterol and triacylglycerols may be influenced by compounds contained in food [111]—it can be assumed that the hypolipidemic mechanism of action of these plants is also similar. It has been suggested that it may be related to the reduction of HMG-CoA reductase (a key enzyme in cholesterol biosynthesis), by the dietary bulb vegetables. This hypothesis may be confirmed by the results obtained by other authors who showed that garlic and its extracts [112,113], as well as onion and its quercetin and its metabolites [55,114], significantly inhibited the activity of this enzyme. Gephard and Beck [115] showed that garlic extracts reduced the synthesis of cholesterol by regulating the activity of the HMG-CoA enzyme. S-allylcysteine and ajoenes also showed a similar ability [27,28,116]. The steroidal saponins present in garlic have also been found to lower total cholesterol and its LDL-C fraction without significant changes in the HDL-C fraction [102]. Initially, allicin was thought to be the active factor responsible for the anti-atherosclerotic effects of garlic. 

However, in vitro studies have shown that water-soluble organic sulfur compounds, mainly SAC (s-allyl-cysteine) in AGE (aged garlic extract) and DADS (diallyl disulphides) in oil, are also potent inhibitors of cholesterol synthesis [27,115]. Moreover, water-soluble compounds have been shown to be less cytotoxic and more efficient in inhibiting cholesterol synthesis than fat-soluble sulfur compounds [27]. According to most authors, quercetin present in onions is the main compound responsible for the regulation of lipid metabolism. Bok et al. [114] showed that supplementation with quercetin in a semi-synthetic diet for rats containing 10 g/kg of cholesterol led to a significant reduction in the absorption of dietary cholesterol, a decrease in blood and liver lipids, as well as positively influencing the activity of liver enzymes. Other studies also showed that bulbs and their extracts decreased the synthesis of fatty acids in vitro and in vivo [26,112], and increased the excretion of cholesterol by increasing the secretion of acid and neutral steroids [117].

**Figure 2:**
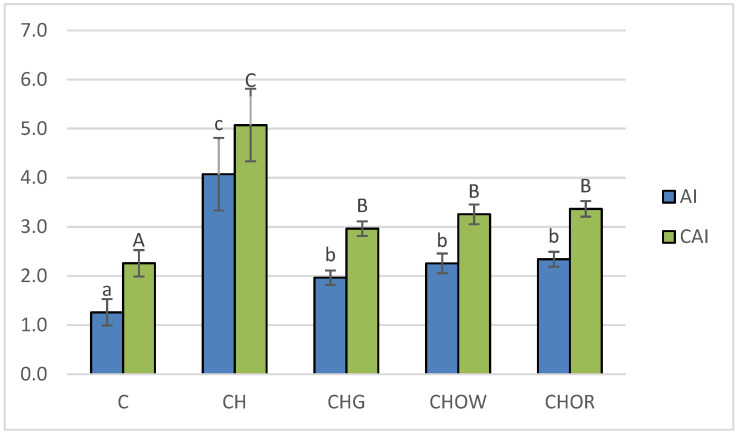
Changes in atherogenic indices (AI and CAI) in rats fed control and cholesterol-containing (1% of cholesterol) diets supplemented with lyophilized vegetables (garlic, white onion, and red onion) during the 28 day experiment. Values are means of 6 measurements ± SD. Means in bars with different superscripts letters in common differ significantly (*p* < 0.05): a–c indicate values for AI and A–C indicate values for CAI. Abbreviations: C—control group; CH—control group with 1% of cholesterol; CHG—group with 1% of cholesterol and lyophilized garlic addition; CHOW—group with 1% of cholesterol and lyophilized white onion addition; CHOR—group with 1% of cholesterol and lyophilized raw red onion addition; AI—atherogenic index, CAI—Castelli’s atherogenic index.

The conducted experiment made it possible to compare the effect of diet with a 1% addition of cholesterol (CH) and the additives used in freeze-dried vegetables on atherogenic indices (AI and CAI) in the blood of rats, and the obtained results are presented in Figure 2. 

The lowest (*p* < 0.05) atherogenic indices (AI and CAI) were found in the control group (C), where they were 1.3 ± 0.3 and 2.3 ± 0.3, respectively. These were over 3.2 and 2.2 times lower than in the atherogenic (CH) diet, respectively, where they were 4.1 ± 0.7 (AI) and 5.1 ± 0.7 (CAI). In animals receiving the addition of freeze-dried onion vegetables in the atherogenic diet, there was a significant (*p* < 0.05) improvement in atherogenic indices. The greatest impact was exerted by the supplementation of the CH diet with garlic, reducing the AI and CAI indices by approx. 51.6 and 41.4%, respectively, in relation to the CH group. White onion supplementation had a slightly weaker effect, as it lowered the atherogenic indexes by approx. 44.5 and 35.7%, respectively, compared to the CH group. Supplementation of the atherogenic diet with the addition of freeze-dried red onion improved the atherogenic indexes of rats to the smallest, but still significant (*p* < 0.05) degree (by approx. 42.5 and 33.5% for AI and CAI, respectively) compared to the CH diet. The results obtained in this study are consistent with the literature data. Studies show that garlic used in the form of various preparations and in various doses (1-4% in the diet) significantly decreased blood lipid indices of rats or mice [103,105]. The main place where cholesterol is formed is the liver, and its synthesis requires considerable energy consumption; therefore, about a third is taken from food. The concentration of cholesterol in the blood depends, among other factors, on its energy value. With an oversupply, the synthesis processes are inhibited, and most of the cholesterol absorbed comes from food. Unused cholesterol accumulates in the bloodstream; as a result, its concentration increases. A large excess of cholesterol over a long period of time can lead to it being deposited in the liver (causing it to become fatty) or in the blood vessels (in the form of plaque). 

The available literature shows that long-term feeding with a diet with high cholesterol content led to a significant increase in lipids, not only in the blood, but also in organs—e.g., in the liver or aorta in rats, mice, rabbits, or hamsters [23,118].

**Figure 3:**
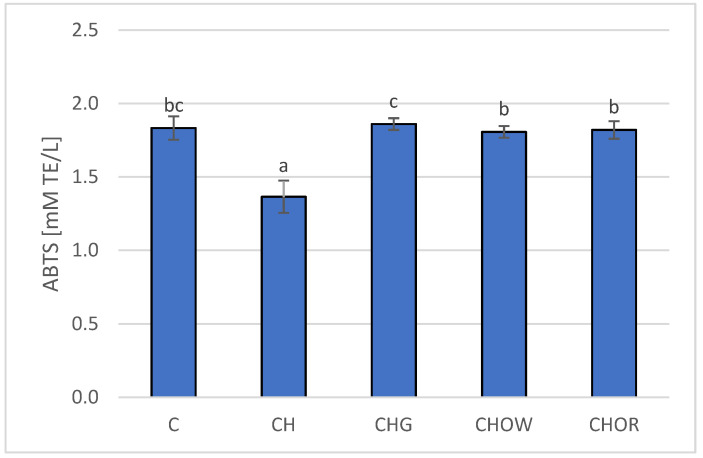
Changes in plasma antioxidant activity (ABTS) in rats fed control and cholesterol-containing (1% of cholesterol) diets supplemented with lyophilized vegetables (garlic, white onion, and red onion) during the 28 day experiment. Values are means of 6 measurements ± SD. Means in bars with different superscripts letters in common differ significantly (*p < 0.05*). Abbreviations: C—control group; CH—control group with 1% of cholesterol; CHG—group with 1% of cholesterol and lyophilized garlic addition; CHOW—group with 1% of cholesterol and lyophilized white onion addition; CHOR—group with 1% of cholesterol and lyophilized raw red onion addition; ABTS—plasma antioxidant activity measured by ABTS test (ABTS—2,2’-Azino-bis-(3-ethyl-benzothiazoline-6-sulfonic acid) diammonium salt).

Increased synthesis of cholesterol and triacylglycerols is also accompanied by an increase in the LDL-C fraction, and its increased sensitivity to oxidation promotes the formation and development of atherosclerotic changes in blood vessels [119]. Therefore, maintaining the balance between oxidation and reduction processes in the body is an important factor in the prevention of these diseases. The available literature shows that consuming garlic may inhibit the oxidation of lipids in the blood [18,19,33,46]. A similar effect was found for onions and for quercetin, which significantly increased antioxidant activity in rats fed a cholesterol diet by increasing the activity of glutathione peroxidase [114]. Therefore, in the research conducted here, the effect of the addition of freeze-dried onion vegetables on the antioxidant activity of plasma in rats fed with an atherogenic diet (with 1% addition of cholesterol) was assessed, and the obtained results are presented in Figure 3.

The research presented here showed that the high-cholesterol (CH) diet significantly (*p* < 0.05) lowered (by approx. 25.7%) the antioxidant potential of rats’ plasma (1.4 ± 0.1 mM TE/L), in comparison with animals fed with the control diet (C) devoid of it (1.8 ± 0.1 mM TE/L); this is also confirmed by research by other authors [18,19,22,33,101]. The addition of freeze-dried onion vegetables to the atherogenic diet significantly (*p* < 0.05) increased the antioxidant potential of rats’ plasma, and these changes depended on the type of additive used. The highest antioxidant potential was found in the plasma of rats fed the diet with freeze-dried garlic (1.86 ± 0.04 mM TE/L) and was slightly lower for the groups supplemented with red and white onions (1.8 ± 0.1 and 1.8 ± 0.1 mM TE/L, respectively). The addition of onion vegetables in the atherogenic diet increased the antioxidant potential of rats’ plasma to the values obtained in the control group (C). 

The suggested mechanism of the influence of *Alliaceae* family plants on the antioxidant potential of rats’ plasma may be based on the direct influence of compounds with antioxidant properties that are contained in these vegetables; alternatively, it can be based on enhancing the activity of antioxidant enzymes present in the organism [18,19,22,33]. The higher antioxidant potential of the body may also protect LDL-C from oxidation better. The confirmation of this hypothesis is reflected in the available literature [18,19,22,33]. Research shows that garlic or onions used in the diet increases the body’s antioxidant potential [18,19,22,33]. Raw garlic homogenate increased (depending on the dose used) the endogenous content of antioxidants and decreased lipid peroxidation in the heart, liver, and kidneys [120]. It inhibited the oxidation of isolated human LDL-C by scavenging peroxides and inhibiting the formation of lipid peroxides [45]. AGE (aged garlic extract) acted as an antioxidant by scavenging the reactive oxygen species [121] and increasing the activity of antioxidant enzymes in vascular endothelial cells [122]. AGE also prevented the reduction of intracellular glutathione (GSH) concentration when incubating endothelial cells with oxidized LDL-C [123]. Due to its high antioxidant activity, onions also play an important role in inhibiting the lipid peroxidation process [124], and the quercetin present in it is considered to be the most important compound with antioxidant properties—responsible for removing reactive oxygen species and increasing the antioxidant potential in the body [18,19,22,33,125].

**Figure 4:**
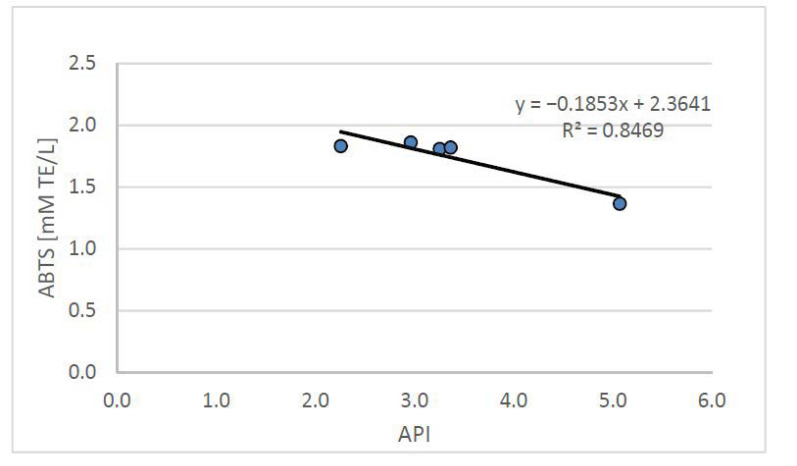
Relation between the API index and plasma antioxidant potential (ABTS) in rats fed control and cholesterol-containing (1% of cholesterol) diet, supplemented with lyophilized vegetables (garlic, white onion, and red onion) during the 28 day experiment. Abbreviations: API—atherogenic index of plasma; ABTS—plasma antioxidant activity measured by ABTS test (ABTS—2,2’-Azino-bis-(3-ethyl-benzothiazoline-6-sulfonic acid) diammonium salt).

Based on the conducted research, it can be concluded that the high antioxidant potential of plasma, which improves the lipid and atherogenic indices of blood plasma, may reduce the risk of developing atherosclerosis and other cardiovascular diseases. The conducted studies showed a statistically significant (*p* < 0.05) negative correlation between the plasma antioxidant potential and the atherosclerotic API index, which is presented in Figure 4. 

The API index, used to define the relationship between blood lipids, allows the determination of the risk of developing atherosclerosis and indicates the need for dietary and/or pharmacological intervention. The API value obtained from the group of rats fed with the atherogenic diet (CH) was, on average, 0.5 ± 0.1 and was significantly (*p < 0.05*) more than three times higher than in the control group (C) (0.2 ± 0.0). The supplementation with the tested freeze-dried onion vegetables used in the high-cholesterol (CH) diet—while increasing the antioxidant potential of the plasma—improved the atherosclerotic index API, which (on average for food groups enriched with onion vegetables) was 0.2 ± 0.0, with the lowest value found in the group supplemented with garlic.

Against the background of the cited data available in the literature, the results obtained in this study confirm the beneficial effects of garlic and onion on the lipid metabolism in the body. Additionally, the results prove that, as a dietary component, these vegetables play an important role in the prevention and alleviation of atherosclerotic diseases. In order to obtain preventive effects, long-term dietary supplementation with garlic and onions is recommended, but this may increase the risk of toxicity of these plants. 

Garlic and onion have been used for centuries as popular aromatic spices in the kitchen and as traditional herbs with medicinal properties, but the side effects of their influence in the body should also be mentioned. Consumption of garlic, especially raw, causes a characteristic smell of the breath or skin, and its excessive consumption may cause a decrease in serum calcium levels, anemia, bronchial asthma, spermatogenesis disorders, or allergic reactions [21,30,41]; one of the most potent examples is DAS (diallyl sulphides) [126]. According to the literature, one of the main irritants in raw garlic is allicin [21,40,126,127]. As emphasized by El-Sheshtawy et al. [126], oil-soluble sulfur compounds are more toxic than water-soluble sulfur compounds—the toxicity of SAC (S-allyl cysteine) to allicin, or to DADS (diallyl disulphides) is 30 times lower. The available literature also shows that excessive consumption of garlic or its preparations may cause gastrointestinal disorders (e.g., diarrhea) [127], as well as causing damage to the intestinal mucosa and stomach, which is attributed to the toxic effect of allicin [128].

In this study, the effect of garlic, white onion, and red onion supplementation on intestinal morphological changes in rats fed a diet with 1% or no cholesterol was assessed, and the results are presented in Table 4.

**Figure 5:**
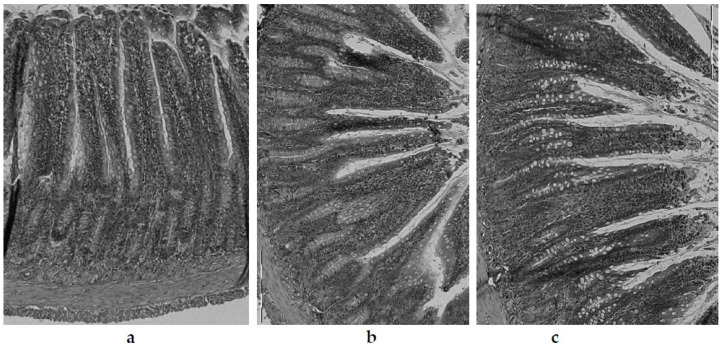
Histological examination of illeum or rats fed with the following diets: control (C) diet (**a**); atherogenic (CH) (containing 1% cholesterol) diet (**b**); and an atherogenic diet, supplemented with lyophilized garlic (CHG) (**c**). Light microscope (×200).

In the experiment performed in this study, the villi length was significantly (*p* < 0.05) highest in the control group (C) (468.6 ± 6.4 µm) (Figure 5a). In animals fed with the atherogenic (CH) diet, the intestinal villi were (*p* < 0.05) more than 22% shorter (363.6 ± 4.9 µm) (Figure 5b). Within the groups of animals fed an atherogenic diet supplemented with freeze-dried vegetables, a clear effect of the additives used on the villi length was found. The most obvious effect of inhibiting intestinal villi growth was seen in the garlic (CHG) supplement group (Figure 5c), where their length was only 305.7 ± 2.1 µm and they were shorter by about 16% compared to the villi recorded in animals receiving an atherogenic diet (CH). In the case of the red onion (CHOR), the inhibitory effect was almost two times smaller (about 9.2%), with the mean intestinal villi length being 330.2 ± 25.9 µm. The addition of freeze-dried white onion (CHOW) used in the atherogenic diet did not have a negative effect on the growth of intestinal villi, their length was, on average, 435.4 ± 16.1 µm and they were significantly (by approx. 19%) longer than in the atherogenic CH group. This suggests that that supplementation with white onion has a positive effect on the growth and development of intestinal villi in rats fed a high cholesterol diet.

A similar relationship was found for the thickness of the tunica mucosa. The highest values (*p* < 0.05) for this parameter were found in animals fed the control group (C) (devoid of cholesterol and freeze-dried vegetables addition) (Figure 5a), where the average thickness of tunica mucosa was 624.5 ± 7.0 µm. Animals fed a diet with a 1% share of cholesterol (CH) (Figure 5b) had a significantly lower value of this parameter (527.7 ± 5.9 µm). The lowest value for the thickness of the tunica mucosa was found in the intestine of rats fed an atherogenic diet with the addition of freeze-dried garlic (Figure 5c) (527.7 ± 6.0 μm), in which the tunica mucosa was almost 12% thinner compared with the atherogenic group, and over 25% thinner compared to the control group (C). The addition of freeze-dried onions used in the atherogenic diet improved the growth and development of the mucosa of the rats’ iliac intestine, and better effects were found for white onions (581.2 ± 14.9 µm) than for red onions (544.3 ± 16.1 µm). For red onions, the mucosa was thicker by approximately 10% and 3% compared to the atherogenic group (not supplemented with onion vegetables).

No such unambiguous differences were found in the thickness of the tunica muscle of the rat, although it should be emphasized that the thickness of the tunica muscle was the highest (*p* < 0.05) in the group of animals receiving an atherogenic diet supplemented with freeze-dried garlic (CHG) (69.1 ± 2.4 µm). The groups of animals supplemented with white and red onion showed an average thickness of the tunica muscle of 66.1± 2.0 µm, but these values did not differ statistically (*p* < 0.05) from those obtained in the atherogenic CH group (67.0 ± 0.5 µm). Moreover, in the case of intestinal crypt depth, no statistically significant (*p* < 0.05) differences were found between the control group (C) and CH, in which the values of this parameter were 130.5 ± 1.8 µm and 135.2 ± 5.2 µm, respectively. The use of freeze-dried onion vegetables in the atherogenic diet significantly influenced the depth of the intestinal crypts only in the group supplemented with garlic (CHG), significantly increasing the value of this parameter to 136.1 ± 4.4 µm.

In the cross-section, the wall of the rats’ illeum intestine is composed of 4 layers—serosa, mucosa externa, submucosa, and mucosa—including intestinal villi visible in the microscopic image as fingerlike, columnar appendages of the membrane, directed towards the lumen of the intestine and Lieberkühn’s crypt, located between the villi bases [74]. In the microscopic image of the intestines of rats from the control group (C) (Figure 5a), the correct structure and contour of the mucosa were preserved, with well-marked cell integrity, without any signs of degeneration or necrosis. The intestinal villi were relatively tall, compact, slender, with enterocytes intact, and with a normal brush border outline.

The intestinal mucosa, due to the digestive and absorbent functions of this part of the gastrointestinal tract, is its most important layer, ensuring the proper absorption of nutrients and their use by the body [74]. According to the literature, impairment of the intestinal lymphatic functions caused by damage, atrophy, or inflammation in the intestinal mucosa may result in impairment of lymphatic functions, and thus, lead to a reduction or delay in body growth [129,130]; this was confirmed in the studies conducted in this paper. Rats fed an atherogenic diet with 1% cholesterol (Figure 5b) showed significantly lower body weight gain (134.9 ± 26.2) compared to animals fed with the control diet (C) (178.2 ± 27.0 g), showing a worse feed efficiency ratio (FER) (3.8 ± 0.6) compared to control diet (3.3 ± 0.3) (Table 3). The available literature lacks data on the effect of a high cholesterol diet on the histological parameters of the rat intestine, although a study by Arija et al. [76] on broiler chickens, fed a diet containing full-fat sunflower seeds, confirms the trend observed in this experiment. The authors showed significant histological changes in the intestinal epithelium of birds fed a high-fat diet, namely the thickening and shortening of intestinal villi, hyperplasia, and vacuolar degeneration of enterocytes, as well as hypertrophy and hyperplasia of goblet cells. In addition, the authors also noted a significant decrease in fat digestibility as well as a decrease in the degree of diet utilization in these animals.

In the present study, the greatest influence on histological changes in the epithelium of the intestine was exerted by the supplementation of the atherogenic diet with freeze-dried garlic (CHG), and in these animals, the damage to the intestinal mucosa was by far the greatest. In the microscopic image (Figure 5c), the intestinal villi showed clear signs of degeneration. The apex of the enterocytes was irregular, protruding from the line of the brush limb protrusion; simultaneously, flattening, erosion and degeneration were visible in these areas. The intestinal villi were markedly atrophied, including blunting, shortening, melting, and significant thickening. The damage to the intestinal mucosa was accompanied by deterioration of growth parameters in rats (Table 3). Animals from this food group (CHG) showed significantly the lowest body weight gain (113.9 ± 27.0 g) and the lowest FER (4.3 ± 0.5 g/g), which confirms the impairment of intestinal lymphatic functions due to damage to the intestine brush border (layers of microvilli that increase the absorbent surface of the intestine). The obtained results are consistent with literature reports, in which the authors attribute the reduction and delay in the growth of animals—or the reduction in dietary utilization rates—to impaired nutrient absorption, which develops as a result of the disappearance of intestinal microvilli, damage to the surface of the brush border, or reduced enzyme production digestive tract [129,130].

However, the available literature lacks data on the effect of supplementation with freeze-dried onion vegetables (garlic, white onions, and red onions) of an atherogenic diet on the histological parameters of the rat intestine. However, there are reports of the effect of using different doses of garlic (50 mg/kg and 100 mg/kg body weight every 2 days for 6 and 12 weeks) in an infected and uninfected *Schistosomiasis Mansoni* mouse model [74]. In these studies, the authors showed mild histological changes in the ileum of mice uninfected with *Schistosomiasis Mansoni* and treated with a lower dose of garlic (50 mg/kg body weight). At twice the dose (100 mg/kg b.w.), they found significant damage to this part of the mucosa, including clear flattening of the tops of enterocytes, or degeneration of intestinal villi. Additionally, in the study of electron microscopy of enterocytes, researchers have shown enlargement of the rough endoplasmic reticulum vesicles, detachment of ribosomes from it, unclear mitochondria, or an increase in the number of lysosomal vesicles. In contrast, in mice infected with *Schistosomiasis Mansoni*, the authors observed an improvement in the histological image of the ileum of mice treated with a lower dose of garlic (50 mg/kg b.w.). At a dose twice as high (100 mg/kg b.w.), they observed a number of pathological changes in the microscopic image of the intestines. Among these, they mentioned intestinal villi atrophy, stromal inflammation, swelling and inflammatory infiltration of the lamina propria, disorders of epithelial and goblet cells, as well as swelling and hypertrophy of the intestinal crypts and muscle membranes. In electron microscopy, they revealed significant damage to the microvilli, hyperactivation of the Golgi apparatus, dilatation of the rough endoplasmic reticulum, edema of the mitochondria, damage to mitochondrial combs, and an increase in the number of lysosomal vesicles.

In this study, the deterioration of parameters within the intestinal mucosa was also accompanied by a marked hypertrophy of the thickness of the tunica muscle and growth of goblet cells and Lieberkühn’s crypts (Table 4). In the intestinal lumen, it was possible to observe the presence of amorphous material, containing mucus or cell fragments, probably the result of inflammation within the mucosa and crypts, as well as necrosis and shedding of some cells. Similar observations were made by Riad et al. [74] in schistosomatic mice treated with a high dose of garlic (100 mg/kg body weight, every 2 days for 6 and 12 weeks). This study and other studies confirm the results of the potentially toxic effects of high concentrations of garlic in the diet, especially if used for a long time, obtained in the study. 

Nakawaga et al. [66] demonstrated the toxicity of crude garlic extract administered to rats at a dose of 5 mL/kg, even leading to death of the animals due to severe gastric damage. Other researchers analyzed the effect of high doses of aqueous garlic extract and garlic oil on changes in organs such as the liver, stomach, lungs, and heart, showing significant overload and congestion [65]. In turn, in studies on the therapeutic properties of plants from the Alliaceae family, Augusti [131] showed that the long-term feeding of rats with high doses of raw garlic caused significant weight loss and reduction or inhibition of rat growth, caused by large anemia resulting from erythrocyte lysis. In turn, Kodera [129] showed that only the allicin contained in garlic can cause damage to the intestinal mucosa and stomach. Hemmaid and Rahmy [132] found degenerative changes, erosion, and necrotic foci in the stomach wall in a study on rats fed a high garlic powder diet (70 mg/kg raw garlic). Amagase et al. [127] showed that raw garlic juice (0.5 mL) used in diets for rats caused significant damage to the epithelium after 2 h, and ulceration and bleeding in the intestinal mucosa after 24 h. They also evaluated three commercially available garlic preparations (capsules) at doses of 133, 108, and 60.5 mg/rat, respectively. They showed that each of them caused serious damage to the duodenal mucosa after 2 h. These researchers suggest that encapsulated preparations of powdered garlic, which protect allicin from degradation in the digestive tract, may be hazardous to the digestive tract. In the available literature, no data were found on the effects of either white or red onions on morphological changes in the intestine of rats fed on an atherogenic diet.

## 4. Conclusions

Extensive research on plants from the *Alliaceae* family proves that garlic and various onion varieties have a high content of biologically active compounds and a high antioxidant potential; therefore, as a dietary component, they play an important role in the prevention and treatment of many disorders, especially cardiovascular diseases. The tested *Alliaceae* vegetables (i.e., garlic (*Allium sativum* L.) and white and red onion (*Allium cepa* L.)) are characterized by a high content of total polyphenols (from 19.4 ± 1.1 to 29.7 ± 1.2 mg GAE/g d.m.), flavonoids (from 3.4 ± 0.31 to 4.0 ± 0.3 mg CE/g d.m.), flavanols (from 4.6 ± 0.4 to 6.7 ± 0.4 µg CE/g d.m.), and anthocyanin (LQQ to 460.2 ± 10.9 mg CGE/kg d.m.). The contents of the components’ bioactive substances change in the following directions: G < OW < OR for total polyphenols and anthocyanins; G < OR < OW for flavonoids; OW < OR < G for flavanols. The high content of bioactive ingredients with antioxidant properties was accompanied by a high antioxidant potential, while the significantly highest ability to deactivate the synthetic cation radical ABTS was characterized by red onion (36.5 ± 6.5 µM TE/g d.m.). Therefore, vegetables from the *Alliaceae* family are an important component of the diet, playing an essential role in the prevention and treatment of many civilization diseases, including atherosclerosis.

Many factors underlie the pathogenesis of atherogenic changes that lead to the development of atherosclerosis, among which, the high concentration of triglycerides, total cholesterol and its LDL fraction, and oxidative stress conducive to their oxidation seem to play an important role in both the initiation and progression of the disease. Therefore, the main preventive factor is the improvement of blood lipid indices and the reduction of oxidative stress, favoring the oxidation of lipid fractions and a general increase in the body’s antioxidant potential. As results from this study show: garlic and onion in freeze-dried form—counteracting numerous risk factors—reduce the possibility of cardiovascular diseases. By lowering the content of lipids and cholesterol and increasing the antioxidant potential of the body, they protect lipids (especially LDL-C) against oxidative modifications and play an important role in the prevention of many diseases, including atherosclerosis.

In the context of atherosclerotic lesions, the preventive or therapeutic effects of consuming vegetables from the *Alliaceae* family are associated with the need for long-term supplementation of the diet with garlic or onion, which in turn may cause the toxic effects of the ingredients contained in these vegetables to occur in the body. No studies have been found in the available literature on the effect of freeze-dried vegetables from the *Alliaceae* family on the morphological parameters of the intestine in an atherogenic diet. As shown in the present study, the use of the addition of freeze-dried onions in the atherogenic diet improved the growth and development of the mucosa of the rats’ iliac intestine (with better results for white onions than red onions). The supplementation of a high-cholesterol diet with freeze-dried garlic significantly damaged the intestinal mucosa in atherogenic rats. Damage to the intestinal mucosa was accompanied by the deterioration of growth parameters in rats and significantly worse use of the diet, which was probably due to impaired lymphatic functions of the intestine.

As it results from the research in this paper, as well as the available scientific literature, it can be concluded that bulbous plants from the *Alliaceae* family have a number of beneficial health or prophylactic effects in various metabolic disorders. However, in order to achieve the desired effects, long-term use is usually advisable; however, especially in the case of garlic, long-term use may be associated with a cytotoxic effect. Therefore, further research in this area is necessary to determine the real and comprehensive benefits associated with supplementation with plants from the *Alliaceae* family (i.e., garlic and various varieties of onions (white and red)), in order to determine the safety of their use. This research is necessary not only in selected, single physiological processes (such as anti-atherosclerotic action), but also to establish their comprehensive effect on the body.

## Figures and Tables

**Table 1:** Bioactive compounds (total polyphenols, flavonoids, flavanols, and anthocyanins) contents and antioxidant activity (ABTS) of plants from the *Alliaceae* family (garlic, white onion, and red onion).

Sample	Total Polyphenols [mg GAE/g d.m.]	Flavonoids [mg CE/g d.m.]	Flavanols [µg CE/g d.m.]	Anthocyanins [mg CGE/kg d.m.]	ABTS [µM TE/g d.m.]
G	19.4 ± 1.1 ^a^	3.4 ± 0.3 ^a^	6.7 ± 0.4 ^c^	LQQ	47.7 ± 1.7 ^a^
OW	24.5 ± 1.2 ^b^	4.0 ± 0.3 ^b^	4.8 ± 0.4 ^a^	28.3 ± 1.3 ^a^	50.9 ± 3.0 ^b^
OR	29.7 ± 1.3 ^c^	3.8 ± 0.3 ^b^	5.9 ± 0.4 ^b^	460.2 ± 10.9 ^b^	69.9 ± 3.1 ^c^

Values are means of 6 measurements ± SD. Means in columns with different superscripts letters in common differ significantly (*p* < 0.05). Abbreviations: G—lyophilized garlic; OW—lyophilized onion white; OR—lyophilized onion red; GAE—gallic acid equivalent; CE—catechin equivalent; d.m.—dry matter; CGE—cyanidin-3-glucoside equivalent; TE—Trolox equivalent; ABTS—2,2’-Azino-bis-(3-ethyl-benzothiazoline-6-sulfonic acid) diammonium salt); LQQ—limit of quantification (may have a footer).

**Table 2:** Comparison of feed intake, cholesterol, and lyophilized vegetables (garlic, white onion, and red onion) intake in rats fed the control and the cholesterol-containing (1% of cholesterol) diet during the 28 day experiment.

Group	Feed Intake [g]	Cholesterol Intake [g]	Lyophilized Vegetables Intake [g]
C	589.8 ± 68.4 ^b^	-	-
CH	501.3 ± 36.3 ^a^	5.0 ± 0.4 ^a^	-
CHG	480.9 ± 42.0 ^a^	4.8 ± 0.4 ^a^	578.6 ± 50.5 ^a^
CHOW	514.0 ± 16.2 ^a^	5.1 ± 0.2 ^a^	621.0 ± 22.4 ^a^
CHOR	578.9 ± 16.3 ^b^	5.8 ± 0.2 ^b^	700.1 ± 23.2 ^b^

Values are the means of 6 measurements ± SD. Means in columns with different superscripts letters in common differ significantly (*p < 0.05*). Abbreviations: C—control group; CH—control group with 1% of cholesterol; CHG—group with 1% of cholesterol and lyophilized garlic addition; CHOW—group with 1% of cholesterol and lyophilized white onion addition; CHOR—group with 1% of cholesterol and lyophilized raw red onion addition.

**Table 3:** Influence of lyophilized garlic, white onion, and red onion addition on performance (body weight gain, FER, and somatic index of liver (SI-L)) in rats fed the control and the cholesterol-containing (1% of cholesterol) diet during the 28 day experiment.

Group	Body Weight Gain [g BW]	FER [g/g BW]	SI-L [% BW]
C	178.2 ± 27.0 ^b^	3.3 ± 0.3 ^a^	3.6 ± 0.4 ^a^
CH	134.9 ± 26.2 ^a^	3.8 ± 0.6 ^ab^	3.7 ± 0.4 ^a^
CHG	113.9 ± 27.0 ^a^	4.3 ± 0.5 ^b^	3.3 ± 0.3 ^a^
CHOW	134.3 ± 12.1 ^a^	3.8 ± 0.3 ^ab^	3.3 ± 0.3 ^a^
CHOR	175.4 ± 17.8 ^b^	3.3 ± 0.3 ^a^	3.7 ± 0.4 ^a^

Values are the means of 6 measurements ± SD. Means in columns with different superscripts letters in common differ significantly (*p < 0.05*). Abbreviations: C—control group; CH—control group with 1% of cholesterol; CHG—group with 1% of cholesterol and lyophilized garlic addition; CHOW—group with 1% of cholesterol and lyophilized white onion addition; CHOR—group with 1% of cholesterol and lyophilized raw red onion addition; BW—body weight, FER—feed efficiency ratio; SI-L—somatic index of liver.

**Table 4:** Influence of lyophilized garlic, white onion, and red onion additions on performance (body weight gain, FER, and somatic index of liver (SI-L)) in rats fed control and cholesterol-containing (1% of cholesterol) diets during the 28 day experiment.

Group	Villi Length [µm]	Crypt Depth [µm]	Thickness of Tunica Mucosa [µm]	Thickness of Tunica Muscle [µm]
C	468.6 ± 6.4 ^e^	130.5 ± 1.8 ^a^	624.5 ± 7.0 ^d^	60.2 ± 0.2 ^a^
CH	363.6 ± 4.9 ^c^	135.2 ± 5.2 ^ab^	527.7 ± 5.9 ^b^	67.0 ± 0.5 ^bc^
CHG	305.7 ± 2.1 ^a^	136.1 ± 4.4 ^b^	465.2 ± 7.4 ^a^	69.1 ± 2.4 ^c^
CHOW	435.4 ± 16.1 ^d^	131.2 ± 3.0 ^a^	581.2 ± 14.9 ^c^	65.9 ± 2.5 ^b^
CHOR	330.2 ± 25.9 ^b^	133.5 ± 2.9 ^a^	544.3 ± 16.1 ^b^	66.3 ± 1.7 ^bc^

Values are the means of 6 measurements ± SD. Means in columns with different superscripts letters in common differ significantly (*p < 0.05*). Abbreviations: C—control group; CH—control group with 1% of cholesterol; CHG—group with 1% of cholesterol and lyophilized garlic addition; CHOW—group with 1% of cholesterol and lyophilized white onion addition; CHOR—group with 1% of cholesterol and lyophilized raw red onion addition.

## Data Availability

Not applicable.
